# Role of Chaperone-Mediated Autophagy in Ageing Biology and Rejuvenation of Stem Cells

**DOI:** 10.3389/fcell.2022.912470

**Published:** 2022-06-28

**Authors:** Emanuela Vitale, Sadia Perveen, Daniela Rossin, Marco Lo Iacono, Raffaella Rastaldo, Claudia Giachino

**Affiliations:** Department of Clinical and Biological Sciences, University of Turin, Orbassano, Italy

**Keywords:** rejuvenation, aging, embryonic stem cells, haematopoietic stem cells, mesenchymal stromal cells (MSC), adult stem cells, pluripotent stem cell, Chaperone-Mediated Autophagy (CMA)

## Abstract

What lies at the basis of the mechanisms that regulate the maintenance and self-renewal of pluripotent stem cells is still an open question. The control of stemness derives from a fine regulation between transcriptional and metabolic factors. In the last years, an emerging topic has concerned the involvement of Chaperone-Mediated Autophagy (CMA) as a key mechanism in stem cell pluripotency control acting as a bridge between epigenetic, transcriptional and differentiation regulation. This review aims to clarify this new and not yet well-explored horizon discussing the recent studies regarding the CMA impact on embryonic, mesenchymal, and haematopoietic stem cells. The review will discuss how CMA influences embryonic stem cell activity promoting self-renewal or differentiation, its involvement in maintaining haematopoietic stem cell function by increasing their functionality during the normal ageing process and its effects on mesenchymal stem cells, in which modulation of CMA regulates immunosuppressive and differentiation properties. Finally, the importance of these new discoveries and their relevance for regenerative medicine applications, from transplantation to cell rejuvenation, will be addressed.

## Background: Stem Cells, Ageing and Autophagy

Stem cells are cells that during their replication cycle can give rise to either stem or differentiated daughter cells. Stem cells are classified into embryonic stem cells (ESCs) and adult stem cells (ASCs) (Young, 2011; De Los Angeles et al., 2015). ESCs derive from the inner cell mass of the pre-implantation blastocyst and are undifferentiated pluripotent cells, having the potential for many different cell fates (Evans and Kaufman, 1981; Young, 2011; De Los Angeles et al., 2015). ASCs are rare populations of cells found in the postnatal organism; they are also undifferentiated cells yet capable of differentiating into limited cell types (Wagers and Weissman, 2004; De Los Angeles et al., 2015). The nucleus of an adult cell can be forced into an altered gene expression pattern to obtain the induced pluripotent stem cells (iPSCs), which are reprogrammed, ESC-like somatic cells (Yamanaka, 2009). ESCs and iPSCs are generally referred to as pluripotent stem cells for their abilities to self-renew and to differentiate into a wide range of cell types (De Los Angeles et al., 2015).

In recent years, much attention has been paid to the processes underlying the regulation of stem cells and the differentiation capacity of cells with particular interest in transcriptional factors, epigenetic modifications and metabolic factors (Ren et al., 2017; Shapira and Christofk, 2020; Sueda and Kageyama, 2020; Ludikhuize et al., 2021). The search for the key to youth is one of the main objectives of these studies. Indeed, if at the beginning it was thought that stem cells were “always young” cells, with an unlimited capacity for self-renewal, it is now known that stem cells are subject to ageing with a consequent decline in regenerative capacity of tissues and organs (Turinetto et al., 2016; Ren et al., 2017; De et al., 2021). Moreover, ageing being associated with a gradual functional decline of organs and tissues, is the highest risk factor for cancer (Zouabi and Bardin, 2020). Demographic change and the ageing of the population have therefore prompted researchers to understand the mechanisms underlying ageing that would allow us to understand the age-related diseases and to identify anti-ageing drugs with the aim of a longer and healthier life.

The link between ageing and the decline in somatic stem cell functions has been known for at least 15 years (Rando and Thomas, 2006; Brunet and Rando, 2007; Hammond and Sharpless, 2008; Lapierre et al., 2015; Bernitz et al., 2016; Kalamakis et al., 2019). Owing to their self-renewal and multi-lineage differentiation properties, during development, stem cells originate the distinct cell populations that compose the different tissues; then they play a key role in maintaining homeostasis and activating repair mechanisms on different tissue levels. However, ageing causes a continuous exposure of stem cells to both intrinsic and extrinsic stress factors, generating cell damage and compromising tissue homeostasis and repair.

Many studies indicate a close correlation between ageing processes and degradation of aberrant and harmful protein accumulations. Damaged proteins can be removed either by the ubiquitin-proteasome system or through autophagy (Vilchez et al., 2014; Llamas et al., 2020; Schaum et al., 2020; Schüler et al., 2020; Kruta et al., 2021).

Autophagy through lysosomal degradation allows the elimination of damaged proteins, cytosolic fractions and cellular organelles. Although autophagy has been extensively studied in the physiology of somatic cells, very little is known about this process in stem cells (Mizushima et al., 2001; Mortensen et al., 2011; Sanchez et al., 2011; Ren et al., 2017; Rastaldo et al., 2020).

It is well-known that mechanisms of self-renewal, differentiation and ageing of stem cells are correlated with the processes of protein degradation. These processes would be responsible for the degradation of aberrant protein accumulations harmful for the functionality and homeostasis of stem cells. Fernàndez et al. (2018) showed that genetically modified mice with higher levels of basal autophagy and consequent improved cellular detoxification capabilities exhibited anti-ageing effects, a reduced rate of spontaneous tumour occurrence and an increased life expectancy of 10%. The beneficial effects of autophagy have also been highlighted in mouse models of Alzheimer’s disease in which the increase in autophagy has preserved the function of stem cells in the brain and prevented cognitive decline (Rocchi et al., 2017).

### The Different Types of Autophagy

**FIGURE 1:**
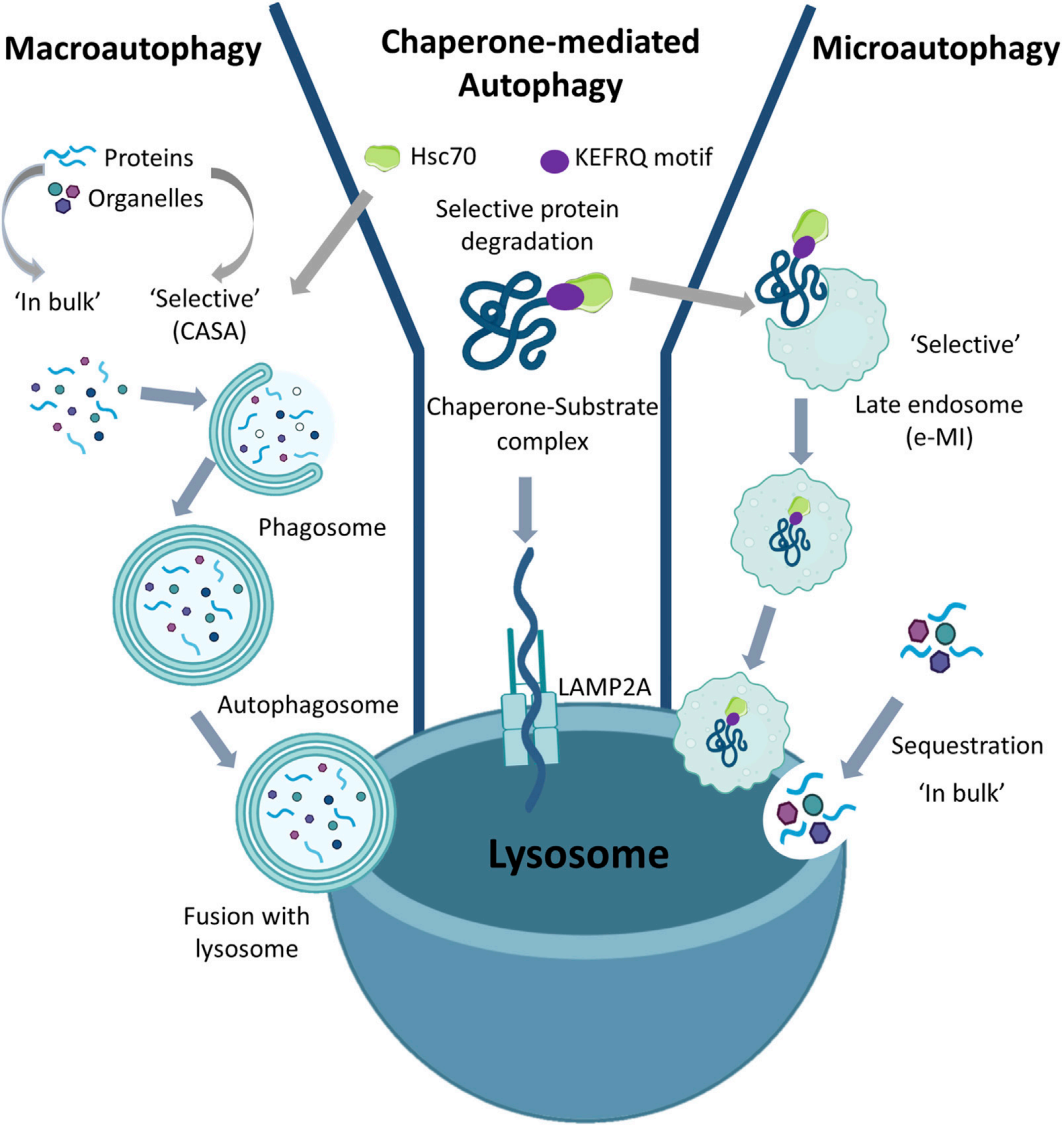
Types of Autophagy: Based on the ways to deliver substrate to the lysosome, autophagy is classified into macroautophagy, microautophagy, and Chaperone-Mediated Autophagy. Macroautophagy can act either in bulk or selective mode. In both selective (in which there may be involvement of Hsc70) and in bulk, the double membrane autophagosomes sequester the substrate (organelles or cytoplasmic proteins) which is internalized in the lysosomes following the fusion of the autophagosomes with the lysosomal membrane. Chaperone-Mediated Autophagy is always selective as in this case only proteins containing the KFERQ motif are recognized by Hsc70 and then transported to the LAMP2A receptor on the lysosomal membrane which allows translocation of the substrate into the lysosome. Microautophagy can also be either in bulk or selective. In bulk microautophagy, cytosolic proteins or organelles can be degraded; in selective microautophagy also called endosomal microautophagy (eMI), the proteins containing the KFERQ motif.

Autophagy is a highly regulated and lysosome-dependent catabolic process aimed at degrading cytosolic components. Based on the different ways of cargo delivery to the lysosomes, autophagy is classified into three types: macroautophagy, microautophagy, and Chaperone-Mediated Autophagy (CMA) (Figure 1).

In macroautophagy the substrates are sequestered within cytosolic double-membrane vesicles termed autophagosomes and delivered to lysosomes through vesicular fusion. Macroautophagy begins with the formation of the phagophore assembly site and continues with nucleation in which the phagophore is formed; then, during the expansion process, the phagophore closes to form the autophagosome (Sotthibundhu et al., 2018). Degradation occurs as a result of fusion of the autophagosome with the lysosome. Alternatively, the lysosomal membrane can invaginate to internalize cargo in vesicles that pinch off from the invaginated membrane. This process known as microautophagy allows for degradation of proteins and organelles sequestered either “in bulk” or in a selective manner (Tekirdag and Cuervo, 2018). In contrast, in the process known as CMA, cytosolic proteins can enter lysosomes for degradation through a protein translocation system which directs them at the lysosomal membrane. In CMA all cargo proteins are selectively delivered to lysosomes upon recognition by heat-shock cognate protein of 70 kDa (Hsc70). Indeed, the target proteins carry a Lys-Phe-Glu-Arg-Gln (KFERQ)-like motif which can be recognized by the chaperone molecules that transport them to lysosomes via the lysosomal-associated membrane protein 2A (LAMP2A) receptor (Kaushik and Cuervo, 2018; Fleming et al., 2022). Until the first CMA studies were performed, autophagy was considered a non-selective form of mass degradation. As only proteins bearing a pentapeptide recognized by Hsc70 in their amino acid sequence could be sorted for CMA-mediated degradation, this demonstrated that the autophagy process can also be selective (Chiang and Dice, 1988).

To date, the landscape of autophagy has changed and it is now known that, besides CMA, selective forms of macroautophagy and microautophagy also exist (Tekirdag and Cuervo, 2018). In all three selective processes the involvement of Hsc70 is evident (Gamerdinger et al., 2011; Tekirdag and Cuervo, 2018). In macroautophagy Hsc70 binds the hydrophobic residues exposed in misfolded or aggregated proteins and under these conditions the degradation process is called Chaperone-Assisted Selective Autophagy (CASA). However, the role of chaperone in CASA is very different from that played in CMA or in a selective form of microautophagy, namely endosomal microautophagy (eMI), where the sequence-mediated targeting of proteins by Hsc70 occurs (Tekirdag and Cuervo, 2018). In fact, during eMI, Hsc70 is degraded by the lysosomal compartment, while during CMA it is returned to the cytoplasm after the substrate has been delivered to the lysosome. Therefore, although Hsc70 is a common chaperone molecule used in selective autophagic processes, its fate can be different. Despite CMA is a clinically still unexplored mechanism, it seems to play a key role in the biology of ageing, arising much interest in researchers. In 2013 Ana María Cuervo highlighted the importance of CMA in various pathological contexts, such as metabolic dysfunctions and neurogenerative pathologies, and she identified a class of molecules capable of stimulating CMA activity by promoting protein degradation (Anguiano et al., 2013). Baharvand et al. (2008) in a proteomic study identified the 60 most expressed proteins in human embryonic stem cells (ESC) undergoing self-renewal, demonstrating that chaperones are among the most involved in this phase. Moreover, this study highlighted the enormous importance of protein quality control systems and CMA for stem cell pluripotency.

This review will focus on the emerging role of CMA in stem cell ageing and its relevant role in stem cell rejuvenation. Furthermore, we will discuss the advantage of targeting CMA as a therapeutic strategy to modulate stem cell function and its possible clinical application.

## Chaperone-Mediated Autophagy Overview

### CMA Mechanism

CMA is an autophagic process capable of selectively removing the material to be degraded without the formation of autophagosomes or vacuoles typical of other autophagic processes (Kaushik and Cuervo, 2018).

**FIGURE 2:**
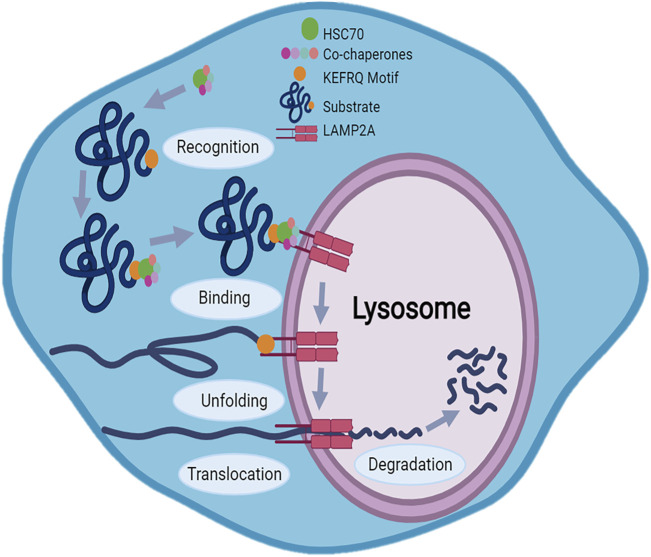
Mechanism of Chaperone-Mediated Autophagy (CMA): CMA mechanism involves five main steps: substrate recognition, lysosome binding, substrate unfolding, translocation and degradation. The KFERQ motif allows the substrate to be recognized by the heat shock protein 70 (Hsc70) which, thanks also to other chaperones, binds chaperone-substrate complex to the cytosolic tails of the LAMP2A receptor on the lysosomal membrane. After unfolding, LAMP2A acting as a transport channel, translocates protein in lysosome where protein degradation occurs.

The mechanism of CMA involves five main steps summarized in Figure 2: substrate recognition, lysosome binding, substrate unfolding, translocation and degradation (Chiang and Dice, 1988; Tekirdag and Cuervo, 2018). During the substrate recognition process, Hsc70 recognizes a specific peptide sequence related to KFERQ motifs in the substrate protein forming chaperone-substrate complex; many other chaperones are involved in this first process, including heat shock protein 90 KD (Hsp90), heat shock protein 40 (Hsp40), Hsp70–Hsp90 organizing protein (Hop), Hsp70-interacting protein (Hip), and bcl2-associated athanogene 1 protein (BAG-1). It was found that Hsp90, Hip, and Hop stabilize the chaperone-substrate complex, while Hsp40 enhance Hsc70 ATPase activity (Kaushik and Cuervo, 2018). The chaperone-substrate complex then binds to the cytosolic tails of the transmembrane receptor LAMP2A, located in lysosomal membrane, which specifically mediates CMA. Since the passage of the substrate protein through the lysosomal membrane requires an unfolded form, the substrate undergoes unfolding with the cooperation of the chaperones. The LAMP2A protein mediates translocation of the substrate acting as a transport channel. Finally, into the lysosomal lumen, lysosomal hydrolase breaks down the unfolded translocated protein in the single amino acids (Chiang and Dice, 1988; Agarraberes et al., 1997; Kaushik and Cuervo, 2018; Salvador et al., 2000; Cuervo and Dice, 1996; Tasset and Cuervo, 2016; Cuervo and Wong, 2014).

### CMA Regulation

**FIGURE 3:**
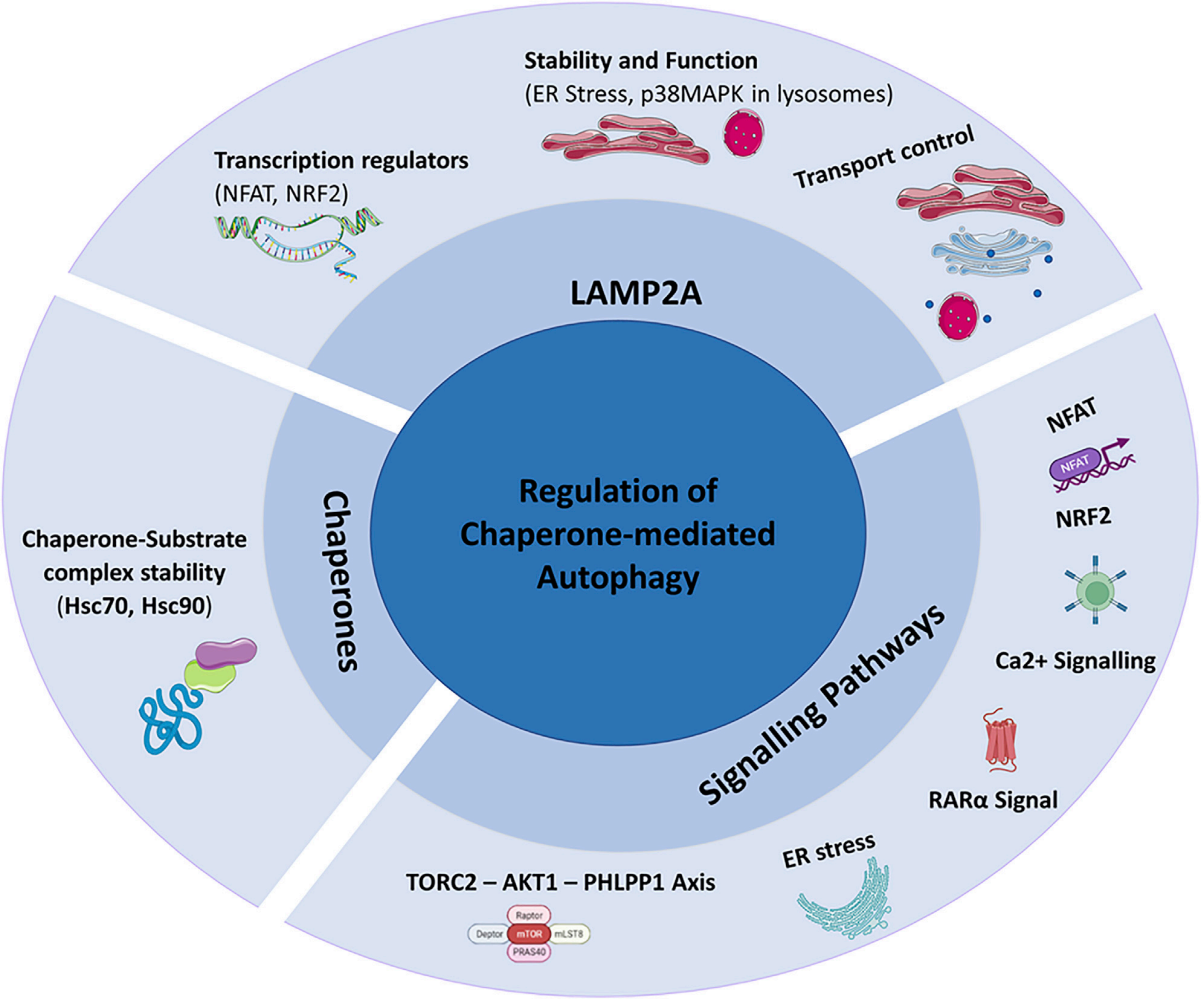
Regulation of Chaperone-Mediated Autophagy (CMA): CMA is mainly regulated by LAMP2A, chaperones and signalling pathways.

CMA is a dynamic process regulated by various factors, among which LAMP2A receptor and chaperone proteins play a main role (Figure 3). The LAMP2A functionality is regulated by direct transcriptional regulation and by protein stability control. Nuclear factor of activated T-cells (NFAT) and nuclear factor erythroid 2-related factor 2 (NRF2) are the main transcriptional regulators of LAMP2A influencing CMA activity (Valdor et al., 2014; Pajares and Ana, 2018). Furthermore, the stability and function of LAMP2A are regulated also by endoplasmic reticulum (ER) stress via the activation of p38MAPK. ER stress-induced chaperone-mediated autophagy (ERICA) is a coupling process between ER stress and CMA. ERICA seems to play an important role in preventing the death of neuronal cells; in fact, the inhibition of ERICA mediated by ER stress and CMA decoupling, would induce the death of neuronal cells in a model of Parkinson’s disease (Li et al., 2019). The activity of the CMA requires LAMP2A transport control, in fact, as demonstrated in cystine disease, CMA activity is reduced when Golgi complex–lysosomal transport pathway of LAMP2A is impaired (Napolitano et al., 2015). Evidence of the important role played by LAMP2A control transport for CMA activity has also been reported in Parkinson’s disease. Here, the absence of Vps35, a protein involved in the endosome-Golgi transport of LAMP2A determines alterations of CMA in dopamine neurons (Tang et al., 2015).

CMA activity is also closely linked to chaperone activity responsible for the formation of the chaperone-substrate complexes, the substrate recognition, unfolding and translocation. The only chaperone known to bind to CMA substrates is Hsc70 (Chiang et al., 1989).

Studies on human B lymphocytes have shown a regulation of Hsc70 mediated by LAMP2C levels, whose overexpression would block the binding between cytoplasmic Hsc70 and substrates of CMA. Hsc70 is also fundamental to promote substrate translocation processes and it is present in the lysosomal lumen (Ly-Hsc70) (Agarraberes et al., 1997).

Hsp90 is responsible for the chaperone-substrate complexes stability and it was shown in primary cardiomyocytes that the mitochondria-related peptide Humanin influences its activity. Hsp90 would promote the link between chaperones and substrates resulting in an increase in cell viability mediated by the regulation of CMA (Gong et al., 2018).

Signalling pathways are among the mechanisms of CMA regulation too and include aforementioned NFAT, Calcium Signalling, ER Stress, RARα Signal and TORC2-AKT1-PHLPP1 Axis (Anguiano et al., 2013; Valdor et al., 2014; Arias et al., 2015; Li et al., 2017; Li et al., 2019).

## Chaperone-Mediated Autophagy in Stem Cells

### Pluripotent Stem Cells

#### Embryonic Stem Cells

Embryonic stem cells are derived from the inner cell mass of early-stage preimplantation embryos. ESCs are able of replicating indefinitely in the absence of senescence and can differentiate into all cell lines. It is still unclear how stemness is preserved in ESCs but it is known that transcriptional and metabolic circuits are responsible for cell fate (Shyh-Chang et al., 2013; Martello and Smith, 2014; De Los Angeles et al., 2015; Chandel et al., 2016; Zhang et al., 2018).

**FIGURE 4:**
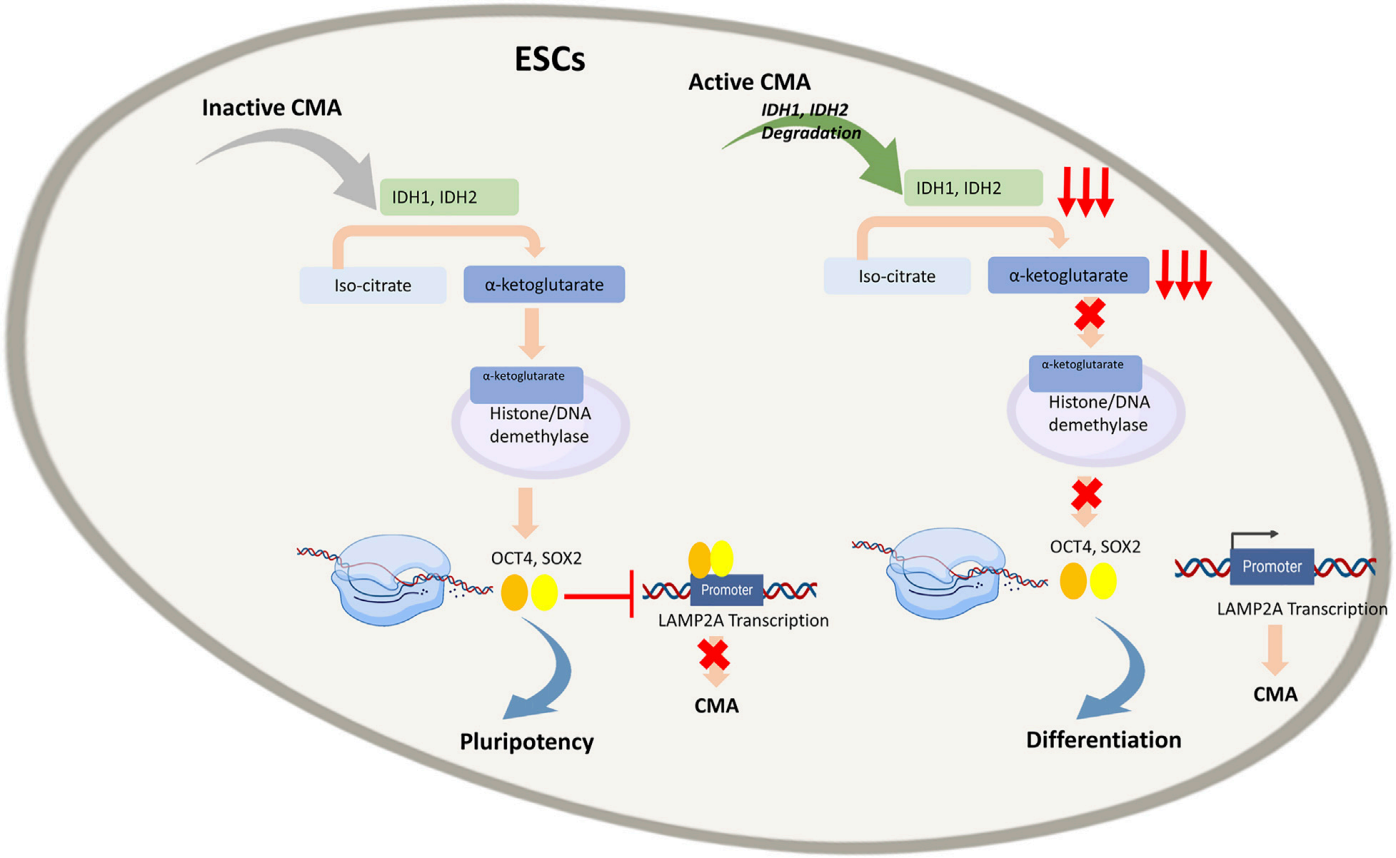
Regulation of Embryonic stem cells (ESCs) by Chaperone-Mediated Autophagy (CMA): In quiescent ESCs, where CMA is inactive, Isocitrate dehydrogenases (IDH)1 and IDH2 convert isocitrate to α-ketoglutarate, a cofactor of numerous histones and DNA demethylases. Through epigenetic control, it enables expression of transcription factors OCT4 and SOX4 that bind to LAMP2A promoter and inhibit transcription of LAMP2A gene and subsequently of CMA that in turn enables to maintain pluripotency of ESCs. In active ESCs, CMA leads to degradation of IDH1 and IDH2, resulting in low α-ketoglutarate levels and consequently low OCT4 and SOX4 expression, enabling LAMP2A transcription. This activates CMA that in turn empowers differentiation of ESCs.

A recent study has shown that low CMA activity promotes ESC self-renewal, whereas its up-regulation enhances ESC differentiation (Figure 4) (Xu et al., 2020). In a mouse model the authors showed that CMA reduces levels of intracellular α-ketoglutarate, an obligatory cofactor for various histone proteins and DNA demethylases involved in pluripotency. These results suggested that CMA can mediate the effect of core pluripotency factors on metabolism, acting as a bridge between the transcriptional and metabolic machinery in ESCs.

Different metabolic states are known to be responsible for mouse ESC (mESC) pluripotency whose high proliferative capacity is closely related to the high activity of different glycolytic enzymes, high glycolytic flux and low mitochondrial oxygen consumption (Kondoh et al., 2007; Wang et al., 2009; Shyh-Chang et al., 2013; Carey et al., 2015; Hwang et al., 2016; Tsogtbaatar et al., 2020). Moreover, metabolic enzymes represent a large pool of substrates for CMA (Schneider et al., 2014; Tasset and Cuervo, 2016). Based on these premises, Yi Xu et al. demonstrated that in genetically modified mESCs characterized by reduced LAMP2A, the presence of many metabolites, including amino acids and nucleotides, was particularly abundant. These data suggested that reduction of CMA flux rises the availability of the anabolic precursors necessary for stem cell proliferation, consistently with the high rate of glycolysis often observed in ESCs (Xu et al., 2020). The authors demonstrated that CMA acts on two enzymes, Isocitrate dehydrogenases (IDH) 1 and IDH2. In the tricarboxylic acid cycle, IDH1 and IDH2 are responsible for the conversion of isocitrate to α-ketoglutarate whose intracellular level plays a key role in maintaining mESCs pluripotency (Carey et al., 2015; Hwang et al., 2016). In fact, α-ketoglutarate is a cofactor of numerous histones and DNA demethylases and consequently, through epigenetic control, it is involved in the pluripotency of mESCs. The authors have shown that the activation of CMA determines the decrease in the intracellular levels of α-ketoglutarate with subsequent loss of pluripotency in mESCs. The correlation between CMA and OCT4 and SOX2 which are known to govern the pluripotency state of ESCs, has been investigated too. Interestingly, OCT4 and SOX2 have been found to inhibit CMA by blocking LAMP2A expression; on the contrary, the increase of LAMP2A expression leads to a reduction in OCT4, SOX2 and an augmented expression of genes associated with differentiation. In addition, the authors tried to propose how CMA can be connected with the pluripotency and differentiation capacity of mESC. LAMP2A, being the receptor for the chaperone-substrate complex in CMA, represents a limiting component for CMA flow. The expression of LAMP2A is closely connected with the differentiation capacity of mESCs as it allows the normal flow of CMA responsible for the degradation of IDH1 and IDH2. Indeed, in quiescent ESCs, CMA is inactive, IDH1 and IDH2 are not degraded and α-ketoglutarate levels are high. Furthermore, it has been shown that it is possible to increase mESC pluripotency by treating cells with cell membrane–permeable dimethyl-αKG (DM-αKG) a precursor for glutamine synthesis. In this scenario, the transcriptional factors OCT4 and SOX2 bind a distal promoter region of the *Lamp2a* gene leading to gene silencing. During cell differentiation, the silencing of SOX2 and OCT4 prevents the binding of the two factors with the *Lamp2a* gene, resulting in an increase in the expression of the protein. This is an elegant example of a bridge between transcriptional, metabolic, epigenetic and cell degradation machinery in which CMA plays a key role in cell fate.

While it was already well-known that ESC proliferation and differentiation are regulated by transcriptional factors and metabolism, it is now evident that CMA links these processes (Xu et al., 2020). CMA is activated in conditions of oxidative stress as demonstrated by the ability of reactive oxygen species (ROS) to promote nuclear translocation of NFAT1 and the consequent enhancement in LAMP2A expression in T cells (Valdor et al., 2014; Kaushik and Cuervo, 2018). Therefore, ROS could stimulate the differentiation process of ESCs.

Pharmacological treatments or genetic manipulation techniques could act at the level of α-ketoglutarate or directly on CMA to control the replicative and differentiation potential of ESCs. However, the previously described study was only conducted on mouse cells (Xu et al., 2020) and it is well known that pluripotent stem cells differ significantly in the regulation of pluripotency between mouse and human (Ernst et al., 2015). For instance, substances that can induce mESCs to differentiate, had either totally different or no effect on the human ones, as well as these cells differ in response to signaling pathways that support self-renewal (Greber et al., 2010). For this reason, it remains to be shown whether these results apply to human embryonic stem cells. Nonetheless, Yi Xu et al. opened the way for new investigations and for the development of new approaches that might be useful to restore the pluripotency lost in degenerative diseases and during the ageing process, as well as to stimulate tissue differentiation for regenerative medicine applications.

#### Induced Pluripotent Stem Cells

The advent of cellular reprogramming has allowed the generation of induced pluripotent stem cells (iPSCs) that can differentiate into virtually any type of somatic cells. Mouse fibroblasts were the first to be successfully converted back to iPSCs through the introduction of four transcription factors: Oct4, Sox2, Klf4 and c-Myc (Takahashi and Yamanaka, 2006; Rademakers et al., 2019). To date, iPSCs can be derived from various differentiated types of human and mouse cells.

Unlike pluripotent ESCs, iPSCs have the advantage that they can be derived from any patient or donor, reducing ethical concerns about using embryo-derived cells. This opens unprecedented perspectives for the generation of autologous donor cells for regenerative medicine purposes, for the development of patient-specific disease models and for the development of new drugs (Shi et al., 2017; Rowe and Daley, 2019).

Although still a subject of debate, in the last decade, some studies have focused on the role of macroautophagy during somatic reprogramming (Xu and Yang, 2022). Instead, there are currently no studies that analyze the possible involvement of CMA. Nonetheless, the differentiation versatility of iPSCs has allowed to investigate the role of CMA in the pathogenesis of neurodegenerative diseases that often involve elderly patients. In this section we will discuss the studies carried out on iPSCs in which the role of CMA has been demonstrated in Parkinson’s disease (PD), which is the first neurodegenerative disorder associated with CMA (Cuervo et al., 2004).

PD affects approximately 2% of over-60 age population and its typical histopathological feature is the presence of intracytoplasmic inclusions in the surviving neurons mainly composed by α-synuclein (Marino et al., 2019).

Mutations occurring in the repeated kinase rich in leucine 2 (LRRK2) are autosomal dominant and manifest as late onset PD. Orenstein et al. showed that in dopaminergic (DA) neurons with LRRK2 G2019S mutation (LRRK2^G2019S^) CMA is impaired by the interaction of α-synuclein with LAMP2A (Orenstein et al., 2013). For this study, the authors differentiated iPSCs in AD neurons starting from healthy individuals and from patients with familial LRRK2^G2019S^ PD. In detail, the authors found high α-synuclein level and a marked LAMP2A-α-synuclein colocalization in LRRK2^G2019S^ patient-derived DA neurons before any obvious signs of neurodegeneration (Sánchez-Danés et al., 2012). Moreover, the authors demonstrated that LRRK2-mediated blockade of LAMP2A multimerization results in accumulation of CMA substrates, including α-synuclein, on the lysosomal membrane surface. In this study, the use of iPSC-derived neurons was particularly advantageous for following the neurodegeneration process from the early stage. Analysis of the data indicated that alterations in CMA are an early event (Orenstein et al., 2013), detectable before manifest neurodegeneration and observed before the previously described macroautophagy impairment (Sánchez-Danés et al., 2012). These results are in agreement with the study of Domenico et al., (2019) where using co-cultures of astrocytes and neurons derived from iPSCs of both healthy donors and PD patients with LRRK2^G2019S^, the authors observed dysfunctional CMA and the accumulation of α-synuclein in PD astrocytes. Furthermore, pharmacological stimulation of CMA in astrocytes had a protective effect due to the increased clearance of α-synuclein.

To note also the study by Kuo et al. (2022) on DA neurons derived from iPSCs that revealed a close link between a set of GBA1 heterozygous mutant (MT) alleles and CMA. The GBA1 gene encodes α-glucocerebrosidase (GCase) and the heterozygous MT alleles of the GBA1 gene are among genetic risk factors for PD. Normally, GCase is folded into the endoplasmic reticulum, transferred to the Golgi apparatus, and delivered to the lysosomes. Using iPSC-derived DA neurons from PD GBA1 heterozygote patients and healthy donors, the authors observed in patient-derived neurons higher levels of MT GCase protein in the cytosol and lower levels in lysosomes. The prolonged binding of MT GCase to the lysosomal surface could disrupts the normal activities of LAMP2A, blocking CMA and leading to α-synuclein the accumulation.

All these studied have allowed to deepen and validate the role of CMA in the neuropathogenesis of PD, giving relevance to the possible pharmacological activation of CMA as a therapeutic strategy for this disease.

### Adult Stem Cells

#### Mesenchymal Stem/Stromal Cells

Mesenchymal stem/stromal cells (MSCs) are multipotent adult stem cells capable of self-renewing or differentiating into different tissues including bone, cartilage, muscle and fat cells, and connective tissue (Friedenstein et al., 1976; Ashton et al., 1980; Owen and Friedenstein, 1988; Han et al., 2019). The commitment of MSCs towards differentiation is strictly regulated by various pathways during development and adulthood (Pittenger et al., 2019).

Demographic changes and an ageing population increasingly push researchers to identify mechanisms to exploit the differentiation capacities of MSCs. This is particularly important for bone tissue where trauma, disease and in particular the advancement of age, lead to tissue degeneration, representing one of the major clinical and socio-economic problems linked to ageing.

**FIGURE 5:**
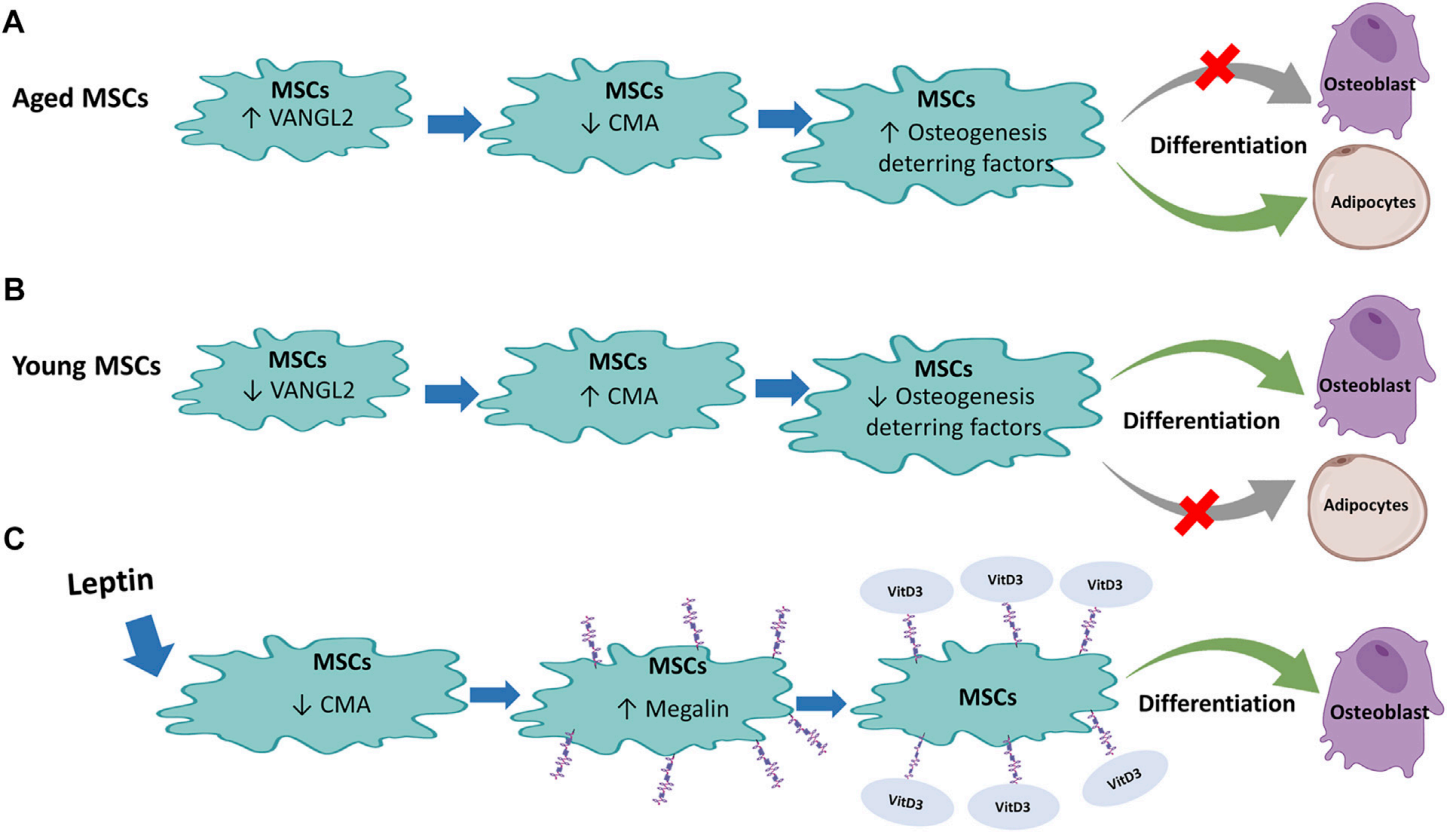
Regulation of Mesenchymal stem cells (MSCs) by Chaperone-Mediated Autophagy (CMA): **(A)** In aged MSCs, active VANGL2 protein decreases CMA with consequent enhancement of osteogenesis-deterring factor level that inhibits osteoblast differentiation and encourages differentiation of MSCs into adipocytes. **(B)** The opposite effect occurs in young MSCs where the low level of VANGL2 and increased CMA induce osteogenesis-deterring factors reduction. This promotes MSC differentiation into osteoblasts and reduce adipocyte differentiation. **(C)** Leptin treatment in rat MSCs decreases CMA that in turn inhibits degradation of megalin, a receptor for vitamin D3. Binding of vitamin D3 to its receptor promotes differentiation of MSCs into osteoblasts.

A group of researchers led by Xiaochun and Zhipeng, studying the regulation of osteogenesis in MSCs, has shown that CMA plays a central role in promoting osteoblastic (OB) differentiation of MSCs (Gong et al., 2021) (Figures 5A,B). Using a mouse model, it was observed that in the OB differentiation phase, MSCs show increased levels of LAMP2A as well as increased CMA activity. An augmented LAMP2A expression was also demonstrated in osteoblasts, indicating the involvement of CMA in OB differentiation even after the initial lineage commitment. Conversely, the observation that low levels of LAMP2A and CMA in MSCs induced to adipocyte (AD) differentiation suggests an inverse correlation between CMA and AD differentiation. The authors demonstrated a compromised osteogenic differentiation ability in bone marrow-derived MSCs and preosteoblasts from elderly people (>70 years) and aged mice (16 months old). To evaluate the association of CMA activity with bone degeneration *in vivo*, LAMP2A expression analysis in femoral sections was carried out. The markedly reduced LAMP2A protein expression in MSCs and preosteoblasts/osteoblasts from elderly people and aged mice in comparison with their young counterparts (for humans, <30 years; for mice, 3 months) suggested that LAMP2A downregulation in MSCs was associated with compromised osteogenic differentiation during ageing *in vivo*. Importantly, a proteomic analysis in men and mice with age-related bone loss allowed to identify the VANGL2 protein, a key regulator of planar cell polarity and organ development (Bailly et al., 2018), as responsible for the mechanism underlying LAMP2A and CMA decrease as well as loss of OB differentiation capacity of aged MSCs. Indeed, VANGL2 expression is dramatically increased in humans and mice with age-related bone loss. In addition, *Vangl2*-deleted MSCs show augmented LAMP2A and CMA levels, with consequent increase in OB differentiation capacity whereas AD differentiation is reduced, thus confirming the role of VANG2L in controlling LAMP2A expression and CMA activity. Furthermore, while OB-induced MSCs are characterized by low VANGL2 levels, its expression is high in AD-induced MSCs. The authors showed that the pro-osteogenic effect mediated by VANGL2 depletion is reversed by LAMP2A down-modulation. Moreover, the *in vivo* study on a genetically modified mouse model has shown that, *Vangl2* deletion in MSCs promotes bone tissue formation while reduces marrow adipose tissue. The study of Gong et al. allowed to understand how CMA controls the commitment of MSCs to osteogenic differentiation. The CMA would, in fact, act by removing osteogenesis-deterring factors. The study of the proteomic variations induced by VANGL2 depletion showed an at least 2-fold reduction of the adipogenic ZNF423 and TLE3 as well as chondrogenic SOX9 and Myd88, which acting downstream to Toll-like or interleukin-1 receptors, would inhibit OB differentiation of MSCs and therefore bone regeneration (Krum et al., 2010; Martino et al., 2016). In detail, it was observed that VANGL2 depletion induces a translocation of adipogenic and chondrogenic inducing factors to LAMP2A positive lysosomes during OB differentiation of MSCs. Conversely, this flux is considerably reduced by LAMP2A depletion. Therefore, the above observations raise the possibility that CMA controls OB differentiation by selective degradation of proteins incompatible with the process. Since the LAMP2A/VANGL2 ratio is high during OB differentiation but low during AD differentiation, the control of this axis could be the key for driving the initial lineage commitment but also for maintaining OB identity during late differentiation in aged patients with all the clinical relevance that this ensues. Moreover, the high bone/marrow fat ratio is correlated with a healthier metabolic condition, and in older patients in addition to bone loss, excessive marrow fat accumulation is often observed. Accordingly, CMA and VANGL2 may represent promising targets in age-related bone loss and the development of a therapeutic strategy that acts on this axis could be useful for the treatment of osteoporosis and other diseases related to bone loss, as well as for metabolic diseases associated with bone/marrow fat imbalance, beyond that for the various applications of regenerative medicine.

At variance with the previously presented scenario, in the same year, He et al. (2021) studied the differentiation capacity of rat MSCs demonstrating an increase in OB differentiation following CMA inhibition (Figure 5C). This result is inconsistent with the study of Gong et al. (2021). However, while the study by Gong et al. was conducted on CMA independent of the master lysosome biogenesis regulator TFEB and of the mTORC2/PHLPP1/Akt axis that, under stress conditions such as serum starvation, induce LAMP2A assembly and consequent CMA activity (Arias et al., 2015), the effects described by He et al. (2021) involved treatment with Leptin, a protein known to be an inhibitor of PI3K/AKT-dependent CMA. It is also known that the MSC OB differentiation is induced by vitamin D whose active metabolite is 1α,25(OH)2D3 (Lips and Van Schoor, 2011; D’ippolito et al., 2002). In MSCs, megalin is among the main receptors of 1α,25(OH)2D3 (Gao et al., 2019). He et al. (2021) proved that leptin treatment stimulates bone differentiation by increasing megalin levels through inhibition of CMA. In particular, leptin decreased LAMP2A and Hsc70 levels and increased AKT phosphorylation. In fact, cells treatment with LY294002, a PI3K/AKT signal pathway inhibitor, induced partial rescue of LAMP2A and Hsc70. Importantly, OB differentiation capacity was also reduced following treatment with PI3K/AKT signal pathway inhibitor, confirming that PI3K/AKT dependent inhibition of CMA is critical to enhancing osteoblast differentiation of rat MSCs induced by the precursor 25(OH)D3. Therefore, this study highlighted another important role of CMA in MSCs OB differentiation, useful for preventing age-related osteoporosis or other pathologies related to bone loss.

Among the physiological mechanisms in which CMA is involved there is the immune response. In fact, CMA participates in the regulation of inflammation and immunomodulation through a variety of mechanisms (Hosaka et al., 2021). For example, NF-κB, a pivotal proinflammatory transcription factor, may be activated by CMA-mediated degradation of the specific NF-κB inhibitor (Cuervo et al., 1998). CMA is also induced in T cells in response to T cell receptor engagement (Valdor et al., 2014).

The extraordinary characteristics of MSCs as unique adult stem cells that have both stem cell properties and immunomodulatory function (Pittenger et al., 2019), prompted us to briefly incorporate in this paragraph the emerging role of CMA in maintaining the immunosuppressive function of MSCs. It is well-known that the inflammatory environment increases the immunomodulatory activity of MSCs (Uccelliet al., 2008; Shi et al., 2017). However, the mechanisms that allow the inflammatory microenvironment to control the immunoregulatory capacity of MSCs are still unclear.

Recently, a study revealed that CMA was inhibited in MSCs in response to proinflammatory cytokines which further indicates that CMA inhibition is a critical contributor to the immunosuppressive function of MSCs induced by inflammatory cytokines, a previously unknown function of CMA (Zhang et al., 2021). In particular, in a mouse model it was shown that treatment of MSCs with proinflammatory cytokines interferon-γ (IFN-γ) and tumour necrosis factor-α (TNF-α) inhibits CMA activity at least in part by activation of AKT with consequent immunosuppressive action. The authors demonstrated that CMA suppression mediated by knocking down LAMP2A in MSCs increased the immunosuppressive abilities of MSCs on T cell proliferation; on the contrary, the increase in CMA through LAMP2A overexpression in MSCs exerted the opposite effect. Moreover, it was demonstrated that CMA orchestrates the immunosuppressive functions of MSCs by reducing chemokine CXC motif ligand 10 (CXCL10) levels which recruits inflammatory T cells, and inducible nitric oxide synthase (iNOS) which leads to the subsequent inhibition of T cell proliferation. The increase in CMA activity and the consequent reduction in CXCL10 levels together with the inability to recruit T cells confirmed the anti-immunosuppressive role of CMA in MSCs. Although the authors have not identified the mechanism of action underlying the results obtained, they hypothesize that the link between CMA and the immunosuppressive functions of MSCs can be traced back to STAT1 and NF-κB pathways (Zhang et al., 2021) which are the two main signalling pathways involved in inflammation-induced expression of CXCL10 (Wilson et al., 2013) and iNOS (Farlik et al., 2010). Indeed, the authors demonstrated that activated STAT1 and NF-κB levels were increased in MSCs with altered CMA and furthermore, inhibition of these two signals by specific inhibitors reduced the levels of CXCL10 and iNOS, while they increased following the inhibition of CMA (Zhang et al., 2021).

The discovery of the role of CMA in the immunomodulatory activity of MSCs is extremely relevant from a therapeutic perspective. Being able to control the immunomodulatory activity of MSCs by acting on the CMA, in fact, could be useful in order to control the action of stem cells in the pathological context, such as autoimmunity or cancer.

Recently, it was proposed that MSCs are able to modulate the macroautophagy of immune and other cells involved in disease pathogenesis (Ceccariglia et al., 2020). In particular, it was shown that MSCs can influence macroautophagy in immune cells involved in injury-induced inflammation, reducing their survival, proliferation and function thus promoting the resolution of inflammation (Chen et al., 2016; Zhu et al., 2016). MSCs can also influence autophagy in adult endogenous or progenitor cells, promoting their survival, proliferation and differentiation by supporting the restoration of functional tissue (Shin et al., 2014; Li et al., 2015; Zhao et al., 2015; Liu et al., 2017; Xiao et al., 2018). The mechanisms by which MSCs can influence the autophagy of other cells are not yet clear but a key role seems to be played by the exosomes released by MSCs (Xiao et al., 2018).

The emerging role of CMA opens up new hints for the study of mechanisms through which MSCs can modulate the autophagy of target cells. Indeed, CMA could influence the therapeutic properties of MSCs providing a broader perspective for the clinical application of MSCs in the treatment of many diseases.

#### Pericytes

Considering the emerging role of CMA in pericytes, we decided to introduce a short section on this cell population whose definition is still ambiguous.

Pericytes are multifunctional cells located within the basement membrane that surrounds capillaries. They can regulate blood flow, are involved in angiogenesis, inflammation and tissue repair as well as regeneration after injury showing stem cell-like properties.

The choice to insert pericytes in a subsection of MSCs derives from one of the definitions of these cells; some authors in fact use the terms “pericytes” and “mesenchymal stem cell” interchangeably, while others argue that pericytes are MSC precursors (Hoshino et al., 2008; Corselli et al., 2010; Nakagomi et al., 2015; Davidoff, 2019; Courtney and Sutherland, 2020). However, the debate on the issue of stemness is still open. Although pericytes share many markers with mesenchymal stem cells, the absence of definitive markers and the heterogeneity of these cell populations makes it difficult to prove their true interconnection.

Pericytes play a key role in immunological defense through the secretion of inflammatory molecules (Valdor et al., 2019). Deregulated CMA can, by modulating their immune response, be associated with pathological processes such as the development of cancer.

Glioma tumour cells have been shown to induce CMA upregulation in surrounding pericytes thus suppressing their antitumour function and inducing immunotolerance to cancer (Valdor et al., 2019; Molina et al., 2022). In detail, tumour-induced aberrant upregulation of CMA in pericytes causes an anti-inflammatory phenotype that prevents T cell activation for tumour clearance. These studies suggest that normalization of pericytic CMA could be an effective antiglioblastoma intervention. In support to this, the authors have indeed shown that the experimental blockade of CMA in pericytes during the glioblastoma-pericyte interaction is sufficient to promote tumour cell death. The authors highlight pericytes as an essential cell type for the establishment of immune evasion in glioblastoma, thus identifying a critical element for the local control of the interaction between tumour cells and pericytes for the down-regulation of the antitumour immune response. Therefore, preventing glioblastoma-induced upregulation of CMA in the pericyte in contact with the tumour cells may represent a targeted strategy for the development of novel therapies against glioblastoma.

Aberrant regulation of CMA occurs with advancing age (Kaushik and Cuervo, 2015) often responsible for an increased risk of malignant transformation. Further investigation on the involvement of CMA not only in stem cells but also in cell populations with immunoregulatory action could open new therapeutic frontiers in the treatment of diseases associated with ageing.

Considering the works examined, we can state that CMA plays a key role in MSCs by acting as a regulator of their differentiation processes and in particular in addressing the bone differentiation of MSCs. Furthermore, the study of the importance of CMA in regulating the immunosuppressive functions of MSCs highlighted a completely new and unexplored function of CMA.

#### Haematopoietic Stem Cells

Haematopoietic stem cells (HSCs) are able to differentiate into cells of the myeloid and lymphoid lineage originating blood cells. By definition, HSCs are also capable of self-renewal and this is a key feature to prevent the bone marrow failing as a result of stem cell pool depletion. Therefore, HSCs are able to pass from the state of quiescence, in which normally they are, to an activated state to originate the blood cells, and then return to quiescence again (Spangrude et al., 1988; Arai et al., 2004; Wilson et al., 2004; Signer et al., 2014). However, the aforementioned characteristics of HSCs can be compromised by ageing, which causes a reduced capacity for self-renewal (Rossi et al., 2007; Beerman et al., 2010; Mohrin et al., 2015; Ho et al., 2017).

Metabolism is known to play a pivotal role in the biology of HSCs. Quiescent HSCs have a glycolysis-based metabolism, while following activation, the fatty acids oxidation within the mitochondria drives the metabolic machine (Simsek et al., 2010; Suda et al., 2011).

Ageing HSCs undergo metabolic reprogramming with an increase in mitochondrial oxidative phosphorylation, in ROS production, in DNA damage and a reduction in the protein degradation system control (Mohrin et al., 2015; Ho et al., 2017).

**FIGURE 6:**
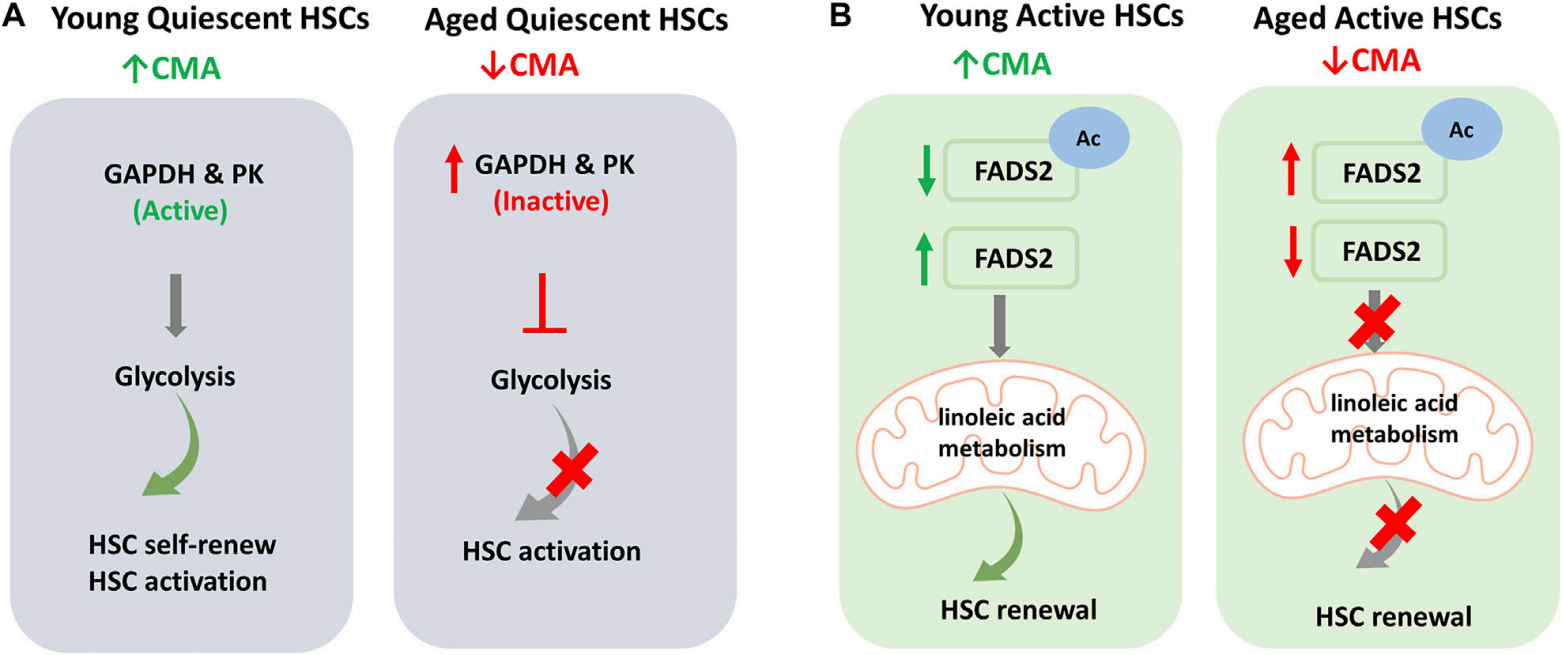
Regulation of Haematopoietic stem cells (HSCs) by Chaperone-Mediated Autophagy (CMA): **(A)** Young quiescent HSCs are characterized by high levels of basal CMA and, thanks to the high activity of glyceraldehyde-3-phosphate dehydrogenase (GAPDH) and pyruvate kinase (PK), have a glycolysis-based metabolism that allows cell self-renewal and activation. In aged quiescent HSCs, reduced CMA activity increases CMA substrates like inactive GAPDH and PK enzymes. This reduces the glycolytic process and consequently limits HSC activation. **(B)** In active HSCs, fatty acids metabolism plays a key role. In young active HSCs the acetylated inactive fatty acid desaturase 2 (FADS2) is removed by CMA and active FADS2 allows linoleic acid metabolism and HSC self-renewal. In aged active HSCs, the reduced levels of CMA results in increase in acetylated inactive FADS2 that inhibits linoleic acid metabolism. This consequently limits renewal capacity of HSCs.

One of the main challenges in studying HSCs is to understand the mechanism of their ageing in order to prevent and/or hinder it. Blocking the ageing process, in fact, could maintain fundamental capacities for the functioning of HSCs, such as self-renewal and differentiation capacity. Dong et al. (2021), using a CMA reporter mouse model showed that the basal activity of CMA was very high in quiescent HSCs derived from young mice compared to older animals; CMA levels were even higher after *in vivo* treatment with 5-fluorouracil (5-FU), a myeloablative agent. Moreover, the study showed that CMA does not affect mature blood cell production; on the other hand, blocking CMA by deletion of *Lamp2a*, reduces HSCs in the bone marrow which, even after treatment with 5-FU, are unable to proliferate. This proved that CMA is dispensable for cell differentiation but is essential to preserve the functionality of HSCs and to prevent depletion of activated HSCs. Furthermore, CMA-deficient cells were characterized by reduced ATP production, increased ROS and accumulation of proteins involved in metabolism, many of which are known to be substrates of CMA. Ana Maria Cuervo and her group have shown that CMA is essential for metabolic machinery regulation in HSCs, playing a leading role in glycolysis and oxidative phosphorylation activation. In fact, in *Lamp2a* deficient HSCs, a reduction of the glycolytic process necessary for quiescent HSCs was observed; in detail, the authors highlighted a reduction in the activity of glyceraldehyde-3-phosphate dehydrogenase (GAPDH) and pyruvate kinase (PK), key enzymes for the glycolytic process and substrates of CMA. Therefore, a low level of CMA, situation that occurs in aged quiescent HSCs, results in the accumulation of inactive GAPDH and PK, preventing glycolysis and HSCs activation (Figure 6A). Moreover, the authors showed that CMA acts also at fatty acids metabolism level. In activated HSCs, CMA is essential for the degradation of fatty acid desaturase 2 (FADS2), the enzyme responsible for linoleic acid metabolism. In fact, the acetylated and inactivated form of FADS2 is among the substrates of CMA in HSCs. Normally, activated HSCs show elevated linoleic and α-linolenic acid metabolism. In contrast, in activated HSCs with blocked CMA, the authors found accumulation of both these precursors. As further confirmation of the link between CMA and fatty acid metabolism, in HSCs with normal levels of CMA, it was possible to reduce the self-renewal capacity by inhibiting the linoleic acid levels regulator, FAD2S. The restoration of the self-renewal capacity in CMA-deficient HSCs after treatment with γ-linolenic acid (GLA), a direct product of FADS2, has proved that HSC functionality is closely linked to α-linolenic and linoleic acid metabolism and to CMA-mediated FADS2 degradation. In detail, Dong et al. have shown that acetylation inactivates FADS2 and triggers its removal by the CMA; this process would therefore be responsible for the balance of the active and inactive enzyme levels, by positively regulating the activity of FADS2 and increasing the metabolism of fatty acids during HSCs activation (Figure 6B). To confirm the role of CMA in HSCs, the evidence that HSCs from CMA-deficient young mice manifested phenotypes similar to HSCs derived from elderly control mice, i.e., altered glycolytic and mitochondrial metabolism, alterations of the proteome as well as reduced CMA activity. Interestingly, the increase in CMA activity through genetic manipulation techniques or drug administration (CMA activators) has improved the functions of the old HSCs. It should be noted that also human CD34^+^ haematopoietic stem cells obtained from elderly donors and treated with CMA activators increased the functional capacities of old HSCs in long-term cultures.

All these discoveries open the way to new horizons, supporting the idea that CMA can be a valid target for HSC rejuvenation as well as the improvement of HSC function in conditions of ageing or stem cell transplantation.

#### CMA in Endothelial Health

Endothelial cells constitute flexible transport networks but have also turned up as critical regulators of stem cell niche function.

In recent years, attention has been paid to the role of autophagy in the health of blood vessel walls (Hua et al., 2022). Macroautophagy has proven important in endothelial cells (ECs) (Torisu et al., 2016), vascular smooth muscle cells (VSMCs) (Grootaert et al., 2015) and macrophages (Ouimet et al., 2011) for maintenance of vasculature homeostasis, in response to lipid challenges and protection against atherosclerosis (Henderson et al., 2021). In contrast, the role of other autophagy types in vascular cells remained less known.

Atherosclerosis is a type of thickening or hardening of the arteries caused by a buildup of plaque in the inner lining of an artery (Benjamin et al., 2017). Among the main risk factors for the development of atherosclerosis there are obesity, hypertension, diabetes and ageing (Benjamin et al., 2017). Atherosclerosis is characterized by vascular endothelial dysfunction that involves ECs activation leading to increased expression of cell adhesion molecules and dedifferentiation of VSMCs from a contractile to an activated secretory and migratory phenotype (Vengrenyuk et al., 2015; Bennett et al., 2016; Wirka et al., 2019).

In 2021, Lei Qiao et al. highlighted the possible role of CMA in atherosclerosis through the regulation of NLRP3 inflammasome (Qiao et al., 2021). In detail, the authors showed a reduction in CMA in advanced atherosclerosis situations both in the aorta of ApoE−/− mice and in human coronary atherosclerotic plaques and an acceleration of the formation of atherosclerotic lesions in mice genetically modified to block CMA by deletion of LAMP2A. The authors also found that LAMP2A deficiency promoted NLRP3 inflammasome activation and subsequent IL-1β release in macrophages and atherosclerotic plaques. On the contrary, the increase in the expression of LAMP2A levels in macrophages attenuated the activation of the NLRP3 inflammasome.

Few months ago, Cuervo et al. showed that blockage of CMA in mice increased their vulnerability to proatherosclerotic challenges, through both systemic and cell-autonomous changes in VSMCs and macrophages (Madrigal-Matute et al., 2022). Loss of CMA in VSMCs promoted their dedifferentiation and higher susceptibility to lipid challenges, while defective CMA in macrophages led to a more proinflammatory phenotype. In detail, the study was conducted on mice fed a pro-atherosclerotic diet by monitoring the activity of CMA in the aortas of animals affected by plaque. After an initial increase in CMA activity in response to the dietary challenge, after 3 months the researchers highlighted a significant accumulation of plaque and the absence of CMA activity in macrophages and VSMCs. The role of CMA for endothelial cell health was confirmed by the presence of almost 40% larger plaques in mice genetically modified to block CMA activity than in WT animals. Consistently, animals genetically modified to upregulate CMA and fed a pro-atherosclerotic diet exhibited smaller plaques and less severity than control animals, as well as significantly improved blood cholesterol levels. Importantly, in their study the researchers provided evidence that weak CMA activity is related to atherosclerosis in humans as well. The authors highlighted that LAMP2A levels in human autopsy-derived atherosclerotic plaques from asymptomatic patients at different plaque stages increase gradually with disease progression; similarly, LAMP2A messenger RNA levels in carotid plaques, surgically retrieved from symptomatic patients, directly correlated with the size of the plaque. In addition, it was shown that after a first ischemic event, patients with higher CMA levels did not have a second stroke; conversely, a second ischemic event occurred in nearly all patients with low CMA activity.

The Cuervo’s study suggests that the level of CMA activity after endarterectomy could help predict the risk of a second stroke and guide treatment, especially for people with low CMA (Madrigal-Matute et al., 2022). It is possible that upregulation of CMA is part of the body’s response to proatherogenic challenges, but factors such as ageing or sustained food pressure, known to inhibit CMA, could reduce the effectiveness of this protective mechanism.

Since CMA levels can be pharmacologically increased in humans, it is possible to speculate on the possibility of substituting carotid endarterectomy with new methods to prevent depletion of CMA levels with ageing.

## Future Clinical Perspectives

Although genetic and epigenetic regulation is at the basis of stem cell identity, increasing evidence supports the control of proteostasis as a major player in stem cell fate by regulating their capacity for self-renewal, pluripotency, and differentiation as well as for the underlying processes of ageing.

Unlike somatic cells, which have a relatively short lifespan, stem cells, due to their ability to either self-renew or differentiate into other cell types, require precise mechanisms for controlling proteostasis to prevent damaged molecules from being “passed down” to daughter cells. Any problems in these control mechanisms can be crucial for stem cells by compromising organism development, stem cell compartment and inducing ageing and related diseases.

In the context of ageing, autophagy stimulation in aged stem cells through genetic and pharmacological approaches has been shown to improve stem cell properties and their regenerative functions. For example, *ex vivo* culture of HSCs derived from human umbilical cord blood in the presence of N- (4-hydroxy-phenyl) retinamide (4HPR) enhanced long-term self-renewal of the cell population (Xie et al., 2019) by increasing the cellular frequency of repopulation after xenograft in immunodeficient mice. Importantly, the 4HPR-mediated improvement on HSC function has been attributed to the activation of proteostasis programs including autophagy. Therefore, the identification of compounds that can increase stem cell expansion and simultaneously preserve their self-renewing functions shows great promise for the future of stem cell transplant approaches and regenerative medicine (Fares et al., 2014; Xie et al., 2019).

If, until a few years ago, the link between autophagy and ageing involved only macroautophagy, recent studies have identified CMA as a protagonist in the processes that regulate the ageing of stem cells, revealing itself as a crucial target in therapies aimed at rejuvenation and at regeneration.

As discussed in this review, CMA plays a key role in stem cells for self-renewal, differentiation, reprogramming and senescence. These new discoveries on both embryonic and adult stem cells pave the way for new possible treatments with potential applications in clinical cell therapy. For example, according to the regulation circuit described by Xu et al. (2020) modulating the CMA would allow to regulate the α-ketoglutarate/succinate ratios by deciding the fate of embryonic stem cells. However, the study by Xu et al. is limited to the use of mouse ESCs which differ significantly from the human model. Furthermore, in the future, it will be appropriate to investigate the possible involvement of other enzymes regulated by α-ketoglutarate. For example, α-ketoglutarate regulates prolyl hydroxylases activity which influence the action of the transcription factor of hypoxia inducible factor 1α (HIF-1α) involved in ESC differentiation. The decrease in α-ketoglutarate could also influence the production of mitochondrial ROS, which play a role in stem cell control (Martínez-Reyes and Chandel, 2020). Moreover, further studies to investigate the possible involvement of other metabolic and non-metabolic enzymes that can be targets of CMA in ESCs would be useful. Interestingly, α-ketoglutarate has been shown to extend lifespan in the nematode worm *Caenorhabditis elegans* in which CMA is conserved (Chin et al., 2014). It is still unknown if this beneficial effect is due to the suppression of CMA, but certainly it might be interesting to investigate this issue in the human being by speculating on the possible integration of α-ketoglutarate to promote a healthy life in humans.

Pharmacological intervention for the regulation of GAPDH and FADS2 levels in HSCs and the control of transcriptional factors essential for differentiation in MSCs would allow, on the other hand, to increase the functionality of stem cells in conditions of ageing or transplantation. Further studies would be interesting to investigate the other metabolic pathways identified as modified in HSCs with defective CMA.

This new evidence designates CMA as a therapeutic goal that, despite completely new and clinically unexplored, will provide promising approaches for the treatment of ageing and diseases related to ageing. In 2013, a class of molecules capable of stimulating CMA was identified. In detail, it was demonstrated that signalling through the retinoic acid receptor α (RARα) inhibited CMA opening the scenario for chemical design aimed at developing synthetic derivatives of trans-retinoic acid to specifically neutralize this inhibitory effect (Anguiano et al., 2013). The action of these compounds has already been tested on mouse models, in which drug treatment has helped to improve the signs of Alzheimer’s disease. Furthermore, as discussed in the section on HSCs, these drugs restored the functionality of the aged haematopoietic stem cells. Furthermore, the discovery of the role of CMA in the immunomodulatory activity of MSC opens new perspectives for the clinical application of MSCs in the treatment of many diseases. For this reason, new studies on the modulation of CMA in cells with immunoregulatory action could be useful for more promising therapeutic approaches.

The advantage of the pharmacological treatment of CMA lies in the intrinsic characteristics of the mechanism itself. In fact, since CMA is based on a highly specific signalling process owing to the KFERQ-like motif recognition on the substrate, its pharmacological manipulation would guarantee a safe and targeted therapeutic intervention respect to the one that targets macroautophagy. Thanks to a targeted degradation, and to the pharmacological modulation of CMA, we might therefore have a successful tool to impact on the fate of stem cells.

## Conclusion

Being able to direct the fate of stem cells is particularly relevant when their pluripotency or differentiation abilities decrease, which occurs during ageing or degenerative disease onset. In these contexts, the possibility to control the decision between maintaining the stem cell pool or inducing cell differentiation is of vital importance. The discovery of the role of CMA in the modulation of these events is therefore very relevant and the possibility of pharmacologically controlling these mechanisms opens a new research horizon in the field of cell rejuvenation. This would be certainly relevant for transplant therapies and regenerative medicine, in particular in damaged tissue restoration, such as bone tissue, a condition that increases with advancing age and the demographic ageing of the population.
